# Supplementary material for: An Untargeted Metabolomics Approach on Carfilzomib-Induced Nephrotoxicity
Source: Molecules. 2022 Nov 16;27(22):7929. doi: 10.3390/molecules27227929 (PMC9697636; doi:10.3390/molecules27227929)
Supplement: Supplementary file 1 [file molecules-27-07929-s001.zip › molecules-2021731-supplementary.pdf]

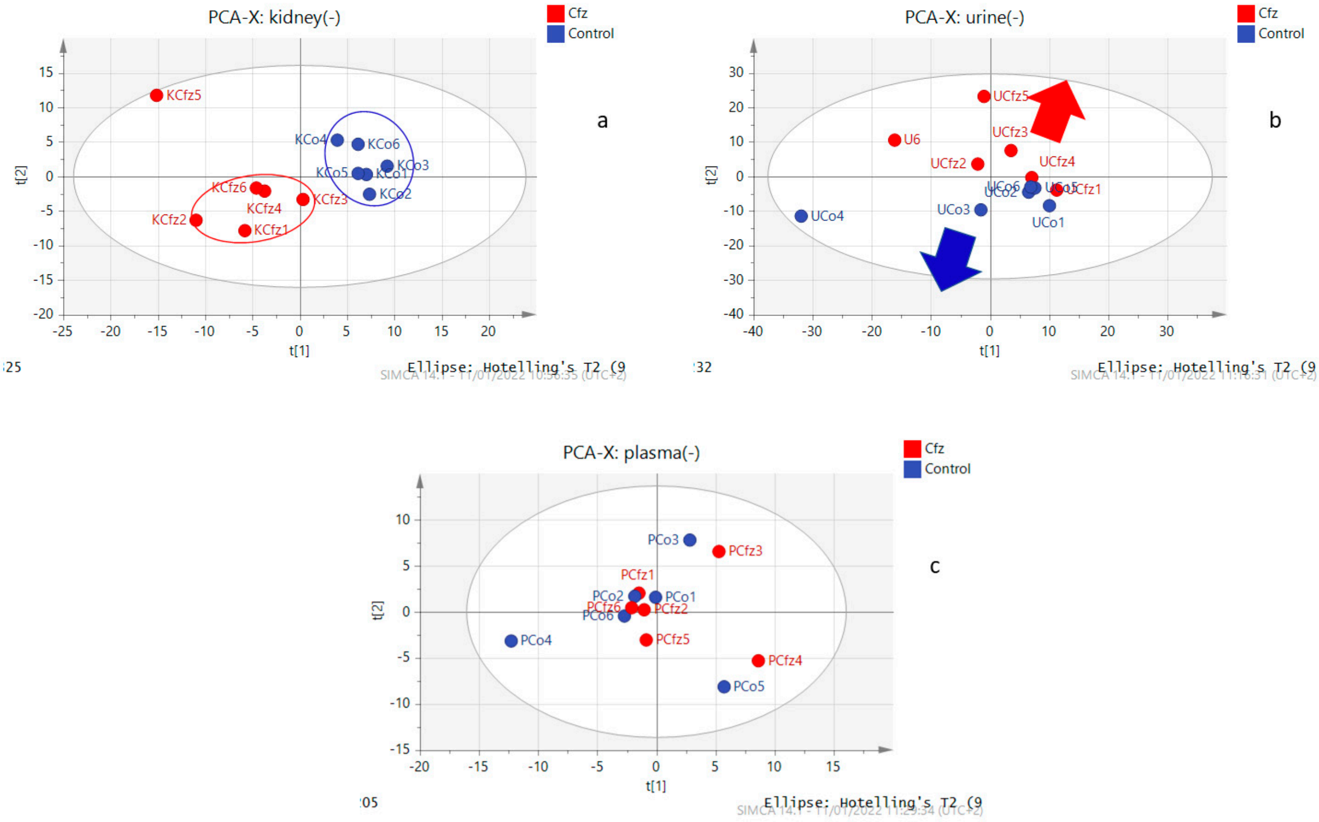

*Figure S1. The PCA scores' plots of the negative-ionization datasets: (**a**) kidney(-), (**b**) urine(-), (**c**) plasma(-).*

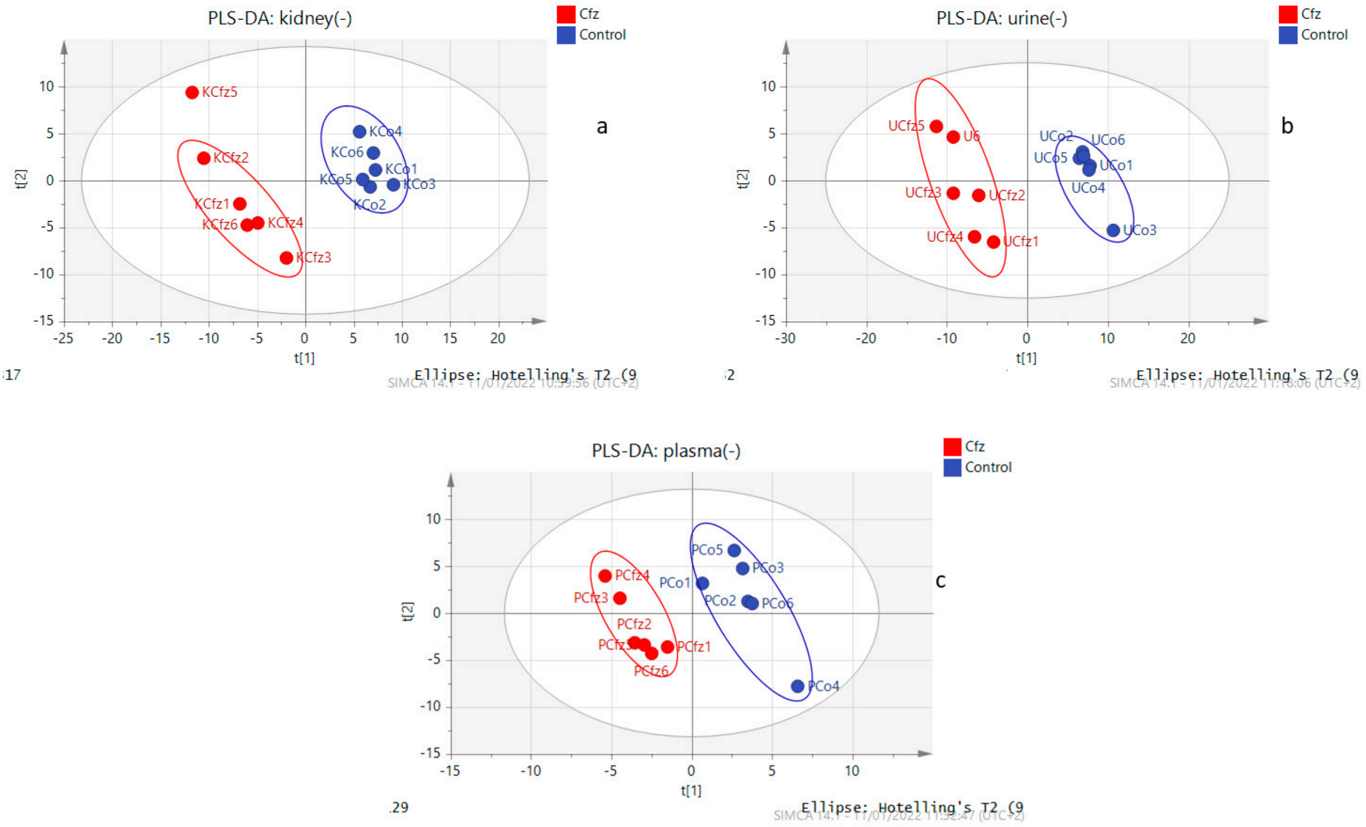

*Figure S2. The PLS-DA scores-plots of the negative-ionization datasets: (**a**) kidney(-), (**b**) urine(-), (**c**) plasma(-).*

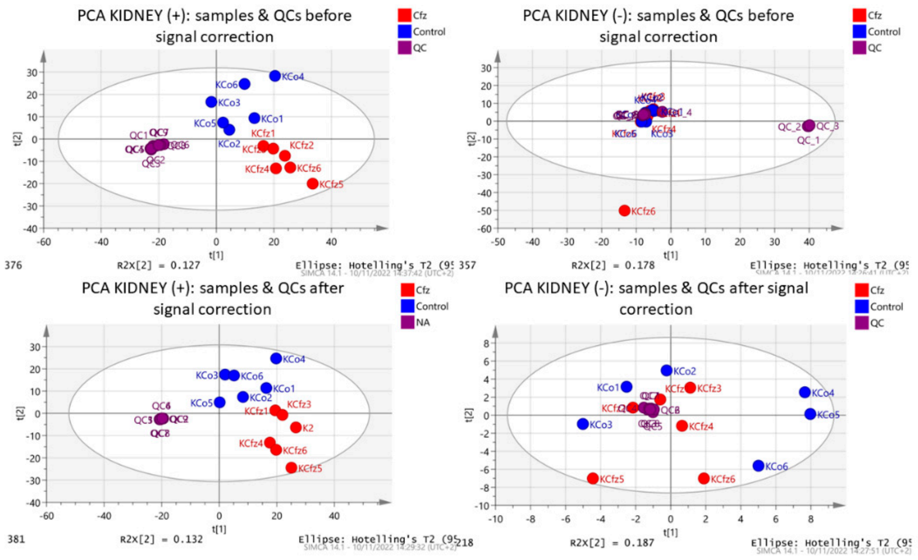

Figure S3. The PCA scores-plots of kidney (samples & QCs) positive and kidney negative dataset before and after the signal correction.

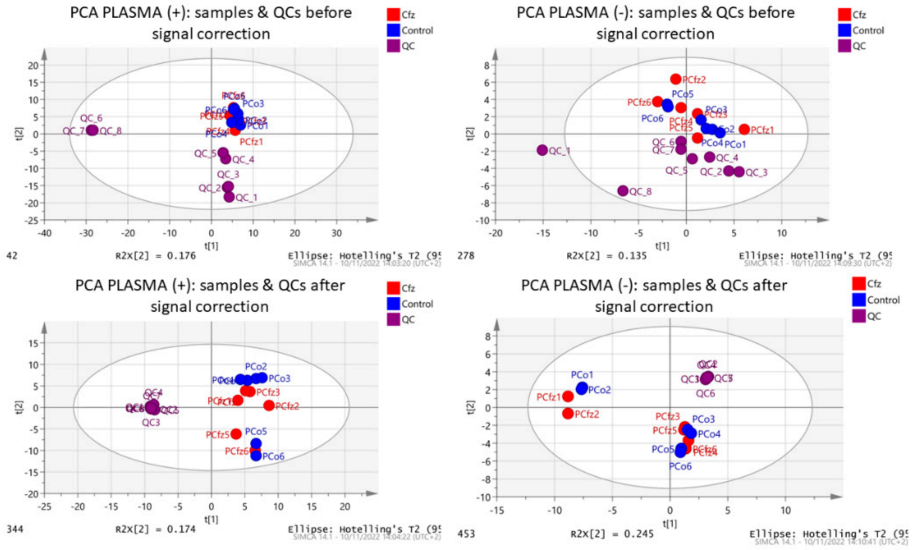

Figure S4. The PCA scores-plots of plasma (samples & QCs) positive and plasma negative dataset before and after the signal correction.

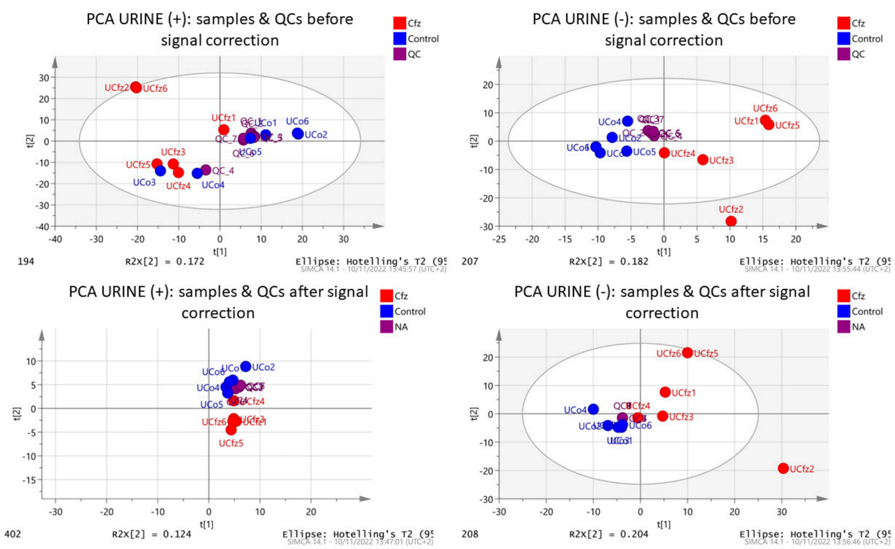

Figure S5. The PCA scores-plots of urine (samples & QCs) positive and urine negative dataset before and after the signal correction.

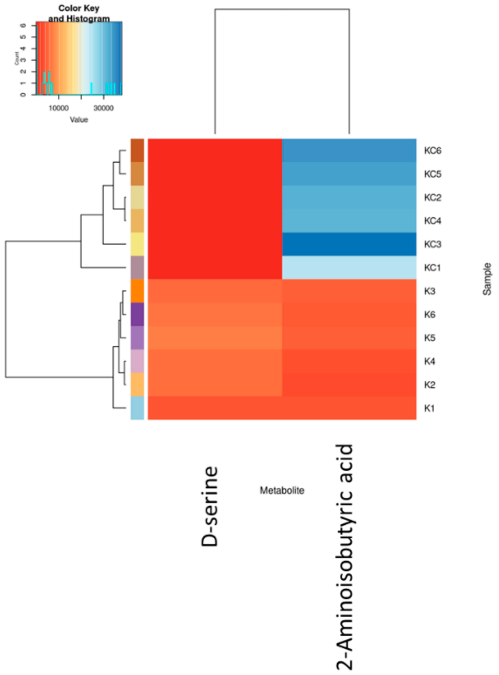

Figure S6. Heatmap description for the correlation of d-serine and 2-aminoisobutyric acid levels regulation in kidney samples of treated (K) and control (KC) mice.
